# Usual Interstitial Pneumonia Pattern and Mycobacteria Lung Diseases: A Case Series

**DOI:** 10.3390/idr17020028

**Published:** 2025-04-03

**Authors:** Maria Angela Licata, Giorgio Monteleone, Enrico Schiavi, Maria Musso, Paola Mencarini, Annelisa Mastrobattista, Serena Maria Carli, Carlotta Cerva, Giacomo Sgalla, Luca Richeldi, Fabrizio Palmieri, Gina Gualano

**Affiliations:** 1 Department of Neurosciences, Sense Organs, and Thorax, Catholic University of the Sacred Heart, 00153 Rome, Italy; mav.licata@gmail.com (M.A.L.); giorgio.monteleone1995@gmail.com (G.M.); enricoschiavi@gmail.com (E.S.); giacomo.sgalla@policlinicogemelli.it (G.S.); luca.richeldi@policlinicogemelli.it (L.R.); 2 Pulmonology Unit and UTIR, Ospedale Civile San Salvatore, 67100 L’Aquila, Italy; 3 Respiratory Infectious Diseases Unit, National Institute for Infectious Diseases “Lazzaro Spallanzani” IRCCS, 00149 Rome, Italy; paola.mencarini@inmi.it (P.M.); a.mastrobattista@inmi.it (A.M.); serena.carli@inmi.it (S.M.C.); carlotta.cerva@inmi.it (C.C.); fabrizio.palmieri@inmi.it (F.P.); gina.gualano@inmi.it (G.G.); 4 Complex Operative Unit of Pulmonology, Department of Neurosciences, Sense Organs, and Thorax, Fondazione Policlinico Universitario A. Gemelli IRCCS, Largo Agostino Gemelli 8, 00168 Rome, Italy

**Keywords:** interstitial lung diseases (ILDs), idiopathic pulmonary fibrosis (IPF), usual interstitial pneumonia (UIP), tuberculosis (TB), non-tuberculous mycobacterial pulmonary disease (NTM-PD)

## Abstract

Background: Interstitial lung diseases (ILDs) are a heterogeneous group of conditions that can cause fibrosis of the lung interstitium, resulting in respiratory failure and death. Patients with an ILD, particularly idiopathic pulmonary fibrosis (IPF) or connective tissue disease-associated ILDs (CTD-ILDs), are prone to develop chronic pulmonary infections such as tuberculosis (TB) and non-tuberculous mycobacterial pulmonary disease (NTM-PD). Methods: This case series examines the management of three ILD patients with a usual interstitial pneumonia (UIP) pattern and concomitant NTM-PD or TB at National Institute for Infectious Diseases “Lazzaro Spallanzani” in Rome, Italy, over three years (2019–2022). Results and Conclusions: Multi-disciplinary discussion (MDD) was crucial to define the therapeutic approach due to the increased risk of side effects and drug interactions. Our work underscored how a comprehensive diagnostic evaluation, enriched by MDD, is useful for optimizing the management and reducing drug-related adverse effects and interactions in ILD patients with cavitary lesions.

## 1. Introduction

Interstitial lung diseases (ILDs) are a rare and heterogeneous group of diseases characterized by a fibrotic overthrow of lung interstitium, which lead to respiratory failure and, finally, to death [1]. Idiopathic pulmonary fibrosis (IPF), the most common and severe among idiopathic interstitial pneumonias, is characterized by the evidence of usual interstitial pneumonia (UIP) pattern in the chest high-resolution computed tomography (HRCT) or in the histopathological examination of lung tissue [2]. Table 1 summarizes the etiologies associated with the UIP pattern.

Non-tuberculous mycobacterial pulmonary disease (NTM-PD) is a rare and emerging chronic infectious disease that tends to be more frequent in patients with underlying pulmonary conditions such as bronchiectasis, cystic fibrosis, chronic obstructive pulmonary disease, and ILDs [3,4]. Similarly to NTM-PD, tuberculosis (TB) represents one of the most common chronic respiratory infections globally that carries a higher prevalence in IPF patients [5]. Although IPF patients are more likely to develop mycobacteria infection, including TB and NTM-PD, detecting these conditions remains difficult due to radiological and clinical findings that may mimic ILD key features, often resulting in misdiagnosis [6,7,8]. Furthermore, the treatment of NTM in IPF patients remains an open challenge due to the risk of side effects and drug interaction between antibiotics and antifibrotic therapy.

Our study aims to present a series of three cases involving patients diagnosed with an ILD with a UIP pattern and concomitant NTM-PD or TB.

**Table 1:** Causes of the usual interstitial pneumonia pattern.

Causes of UIP Pattern	References
Idiopathic pulmonary fibrosis	[1]
Hypersensitivity pneumonitis	[9]
Asbestos exposure	[9]
Sarcoidosis	[10]
Familial pulmonary fibrosis with surfactant genes mutations (SFTPA1; SFTPA2; SFTPC; NKX2.1; ABCA3) or telomere gene mutations (TERT; TERC; RTEL1; PARN; DKC1; TINF2; NOP10; NHP2; ACD; NAF1; ZCCHC8; RPA; POT1)	[11,12]
Drug-induced ILDs	[9,13]
Rheumatoid arthritis	[9,14]
Systemic sclerosis	[9,15]
ANCA-associated vasculitis	[16]
Anti-synthethase syndrome	[9,17]
Hermansky–Pudlak syndrome	[18]

UIP: usual interstitial pneumonia; ILD: interstitial lung disease; ANCA: anti-neutrophil cytoplasmic antibodies.

## 2. Materials and Methods

This investigation was conducted as a retrospective case series study, encompassing patients diagnosed with an ILD and either NTM-PD or pulmonary TB who were admitted to the National Institute for Infectious Diseases “Lazzaro Spallanzani” in Rome, Italy, over three years (2019–2022). Data were retrieved from the electronic medical records of these patients, including demographics, clinical presentation, imaging findings, microbiological results, surgical interventions, and therapeutic regimens.

A supportive literature review was performed to contextualize the findings and enhance our understanding. Relevant research articles exploring the association between IPF and NTM-PD or TB were identified through a systematic search using the PubMed database. The search strategy employed specific keywords, including “NTM-PD”, “ILD”, “UIP”, “IPF”, and “TB”, to ensure the inclusion of studies that directly addressed the intersection of these conditions.

## 3. Case Presentation

Table 2 summarizes the main demographic, clinical, microbiological, and radiological characteristics of the three patients described below in greater detail.

### 3.1. Case #1

**Figure 1:**
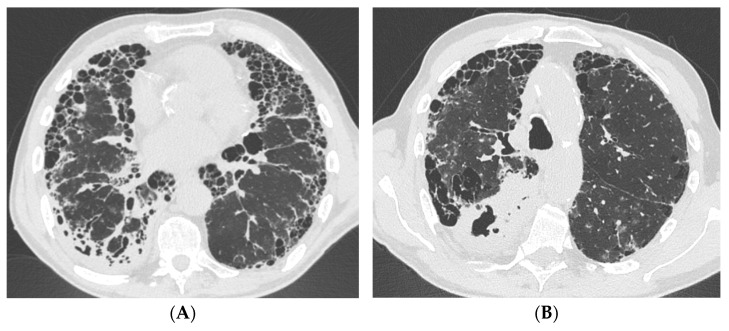
Case #1 contrast-enhanced CT scan acquired in the emergency room during massive hemoptysis. UIP pattern is evident in the lower lobes (**A**), while the most extensive consolidation is located in the apical segment of the right lower lobe (**B**). Abbreviation: CT, computed-tomography; UIP, usual interstitial pneumonia.

A 76-year-old male, a former smoker, with a three-year history of connective tissue disease-associated interstitial lung disease (CTD-ILD) classified as rheumatoid arthritis (RA) with a UIP at chest HRCT, as adjudicated by multidisciplinary discussion (MDD), managed with azathioprine and long-term oxygen therapy, was referred to the Emergency Department with massive hemoptysis. Upon admission to the emergency department, the patient presented with hypoxemic hypercapnic respiratory failure that required oxygen therapy via Venturi mask with a fraction of inhaled oxygen (FiO_2_) equal to 40%. Laboratory tests revealed significantly elevated inflammatory markers and anemia, with a hemoglobin level equal to 8 g/dL, necessitating a transfusion of two units of packed red cells. Therefore, an urgent contrast-enhanced chest CT scan was performed; it demonstrated the presence of UIP pattern and bilateral cavitary lung consolidations, of which the most extensive was located in the right lower lobe (Figure 1). Conversely, no active sources of bleeding were identified. Following hemodynamic stabilization, a bronchoscopy with bronchoalveolar lavage (BAL) was performed to further evaluate the pulmonary findings at chest HRCT; while endobronchial blood clots were removed, no active signs of bleeding were observed. Then, the patient was administered broad-spectrum empirical antibiotic therapy with piperacillin-tazobactam and vancomycin, despite the absence of microbiological results. The bacterioscopic assessment of the BAL fluid tested positive for acid-fast bacilli, while the polymerase chain reaction (PCR) for *Mycobacterium tuberculosis* (Mtb) was negative. Additionally, PCR testing confirmed the presence of *Mycobacterium kansasii*, which was further confirmed by microbiological culture. The administration of antibiotic therapy improved clinical status and gas exchange by reducing FiO_2_ from 40% to 24%. The patient started a targeted antimicrobial therapy with rifampin, azithromycin, and ethambutol, which was well-tolerated, leading to the patient’s discharge with oxygen supplementation only during physical exertion. Symptoms such as persistent catarrhal cough, hemoptysis, low-grade fever, and weight loss gradually resolved after six months of therapy. Hence, the case underwent a second MDD, and although criteria for progressive pulmonary fibrosis (PPF) were identified, antifibrotic therapy with nintedanib was not initiated due to the higher risk of iatrogenic hepatitis associated with concomitant rifampin use. Eventually, antifibrotic treatment was not initiated and deferred until the completion of antimycobacterial therapy, which lasted 12 months from the date of negative sputum cultures, for a total of 18 months.

### 3.2. Case #2

**Figure 2:**
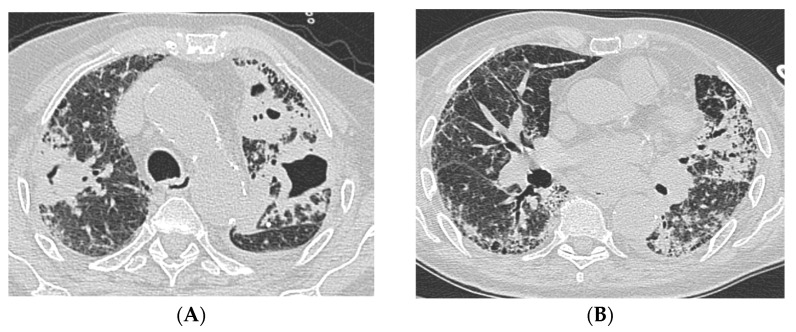
Case #2 chest HRCT scans acquired in the emergency room. Multiple bilateral excavated lesions and parenchymal consolidation with air bronchogram are more evident in the upper lobes (**A**), while the UIP pattern characterized by traction bronchiectasis and reticulation is more prominent in the lower lobes (**B**). Abbreviations: HRCT, high-resolution computed tomography; UIP, usual interstitial pneumonia.

A 82-year-old woman with a history of CTD-ILD secondary to RA and thrombosis of femoral veins was admitted to the Emergency Room for hypoxic–hypercapnic respiratory failure requiring oxygen therapy with FiO_2_ 35% in a febrile state. The patient complained of dyspnea, cough, and weight loss (8 kg) over the previous 3 months. In anamnesis, there was recent close contact with active TB. Laboratory tests revealed elevated inflammatory markers, neutrophilic leukocytosis, moderate anemia, and high D-dimer levels. Chest X-ray showed bilateral reticular diffuse interstitial lung alterations with consolidation in the left upper field. Chest HRCT demonstrated multiple bilateral excavated lesions, parenchymal consolidation with air bronchogram, and a UIP pattern characterized by traction bronchiectasis and reticulation (Figure 2). The patient underwent bronchoscopy with BAL: fluid molecular essay would later result positive for Mtb. Moreover, the mycobacteria growth indicator tube tested positive for TB resistant to isoniazid, rifampin, streptomycin, and pyrazinamide. Therefore, antibiotic therapy with amikacin, amoxicillin/clavulanate, meropenem, clofazimin, cicloserin, bedaquilin, and moxifloxacin was started. During the prolonged hospitalization, the patient received a red blood cell transfusion (two units) for drug-induced anemia. After two months of prescribed antibiotic therapy, a follow-up contrast chest computed tomography (CT) showed the reduction in extension of the excavated lesions and the consolidations and highlighted subsegmental pulmonary embolism. After a cardiological consultation, the patient was finally discharged home with no indication for anticoagulant treatment and no need for oxygen therapy at rest, while maintaining the indication for supplemental oxygen during exertion. The patient died at home the following month, prior to the next scheduled outpatient visit.

### 3.3. Case #3

A 70-year-old male, former smoker, with known contact with active TB presented with productive cough and dyspnea on exertion. The patient was evaluated in an outpatient setting and underwent a chest X-ray, which showed reduced expansion of the left costophrenic sinus and accentuation of the lung pattern. A 10-day empiric course of antibiotics (prulifloxacin) and oral corticosteroids was prescribed; however, this treatment did not lead to any clinical improvement. The patient subsequently presented to the emergency department, where an initial evaluation revealed no respiratory failure but significantly reduced oxygenation levels relative to age. Laboratory tests showed an increased white blood cell count, moderately elevated inflammatory markers, mild anemia, and high ferritin levels. A subsequent chest CT scan revealed no parenchymal consolidations but demonstrated diffuse reticular thickening of the intra- and interlobular septa, more pronounced in the subpleural regions and the posterior segments of the lower lung lobes. Additionally, honeycombing and multiple traction bronchiectasis were observed. Given the persistence of symptoms and a productive cough unresponsive to first-line therapy, an induced sputum sample was obtained. PCR for *Mycobacterium tuberculosis* on the sample was positive.

Antitubercular therapy with rifampin, isoniazid, ethambutol, and pyrazinamide was initiated during hospitalization, with good tolerability and no evidence of hepatic toxicity. After two weeks of treatment, the patient was discharged home. Upon reevaluation in the outpatient clinic the following week for a suspected allergic reaction, pyrazinamide was discontinued and replaced with moxifloxacin. Concomitantly, the case underwent MDD, where the diagnosis of IPF was made. Upon completion of antitubercular treatment for 6 months, antifibrotic therapy with nintedanib 150 mg was initiated, with good tolerability.

## 4. Discussion

Our case series has described three cases of patients with UIP pattern and mycobacteria infections, either concomitant or following the ILD diagnosis.

Certain symptoms commonly associated with pulmonary fibrosis, including shortness of breath, fatigue, progressive dyspnea, and weight loss, can also manifest in cases of pulmonary mycobacteriosis. While dry cough is typically characteristic of pulmonary fibrosis, pulmonary mycobacteriosis is more often accompanied by productive cough. Such overlap in clinical presentations can mask the presence of underlying chronic mycobacterial infections, whose diagnosis relies on further diagnostic investigations [1,3,4].

In patients with IPF, several studies have documented an increased prevalence of chronic pulmonary infections, including nocardiosis, chronic pulmonary aspergillosis, NTM, and Mtb [19,20,21]. In 2012, Park and colleagues reported a significantly higher incidence of NTM-PD in IPF patients compared to the general population. This finding was observed in both patients undergoing treatment with immunosuppressive drugs, which represented the standard therapeutic approach at the time, and in untreated individuals [4]. The authors attributed this elevated risk to structural and anatomical changes associated with the disease, such as the development of honeycombing [4,6,7].

Hwang et al. reported that NTM-PD associated with IPF appears radiologically as lobar or segmental consolidation with or without cavitation, mimicking other pulmonary infections [22]. Similarly, Chung et al. highlighted that TB in individuals with IPF can radiologically resemble other serious conditions, such as lung cancer or bacterial pneumonia, complicating the diagnostic process [6]. Additionally, Fibla et al. conducted a study in which pathogens such as *Histoplasma*, *Nocardia*, and *Aspergillus* were identified through surgical lung biopsies in IPF patients [23]. These findings led to changes in clinical management, underscoring the importance of precise pathogen identification in guiding treatment strategies. Given these complexities, Odashima and colleagues emphasized the need to investigate the causes, the incidence rates, and the risk factors associated with chronic pulmonary infections in the IPF population. Such investigations are vital for improving diagnostic accuracy and tailoring treatment approaches [24].

Cavitary lesions have also been reported in patients with CTD-ILD [25]. Among these, rheumatoid nodules are the most frequent pulmonary manifestation of RA, although they are typically asymptomatic. While cavitation is uncommon, its occurrence can result in severe clinical symptoms due to active disease or concurrent infections [26]. The use of corticosteroids and immunosuppressive agents in managing pulmonary fibrosis related to RA and systemic sclerosis has been associated with an increased risk of NTM infection and related mortality [27,28,29]. Given the potential impact on treatment and prognosis, Yoo and Shu underscored the importance of systematic screening for NTM or TB infections at the time of connective tissue disease diagnosis [3,28]. Such proactive screening measures are crucial, as they can significantly influence treatment decisions and improve patient outcomes.

Oh and colleagues emphasized the need for meticulous follow-up in patients with ILD, as they are at an elevated risk for developing chronic infections or peripheral tumors. In recently diagnosed cases of non-tuberculous mycobacteria-associated interstitial lung disease, the lesions frequently manifest as consolidated cavities within areas of fibrosis or honeycombing [30].

Several studies have reported a notably high incidence of NTM or TB infections in patients with ILD exhibiting a UIP pattern. Most cases involve patients with NTM-PD who present with post-infectious

ILDs often present without a progressive fibrosing course [3,6,7,31]. However, it is worth noting that these investigations were conducted before the implementation of current guidelines that precisely define the radiologic criteria for the UIP pattern [2]. This lack of standardized criteria at the time may have resulted in an overestimation of cases attributed to this pattern.

Recent studies have indicated that in the context of cavitary lung lesions, the use of induced sputum collection may be adequate for the diagnosis of NTM-PD or TB [32,33]. This diagnostic approach is particularly advantageous in settings where bronchoscopy is not readily accessible, providing a less invasive and more feasible alternative for obtaining respiratory specimens. Such findings underscore the importance of optimizing diagnostic strategies to improve accessibility and reduce procedural risks, especially in resource-limited environments.

In a recent review, Juzar Ali highlighted the essential role of a multidisciplinary approach in managing patients with NTM-PD [34]. In our case series, for all patients a multidisciplinary evaluation was important to define diagnosis and, more importantly, to balance antifibrotic and antibiotic therapies. This highlights the need for collaborative decision-making to optimize therapeutic outcomes for these complex cases. The pharmacological management of these infections is certainly an open challenge.

Our case series highlights the necessity for meticulous monitoring and the frequent need for challenging clinical decisions when managing complex treatment regimens in patients with ILD and cavitary lesions or productive cough.

The most recent Clinical Practice Guideline for pulmonary fibrosis in adults recommends pharmacological treatment with either nintedanib or pirfenidone in IPF, while only nintedanib is suggested for the treatment of PPF [2]. Nintedanib is an intracellular tyrosine kinase inhibitor with antifibrotic properties that has shown a manageable tolerability profile in clinical trials in patients with fibrotic ILDs [35]. Gastrointestinal adverse events, especially diarrhea, were the most frequently reported in clinical trials and global pharmacovigilance data. Drug-induced liver injury, while uncommon (31.5 events/1000 PY, with a median time to first onset of 60 days), has been associated with nintedanib [36]. Liver enzyme and bilirubin elevation were typically reversible with dose interruption or reduction. Consequently, liver function tests should be conducted before initiating treatment and at appropriate intervals thereafter [37].

Under these premises, in the three cases reported in our study, priority was given to antimycobacterial therapy over antifibrotic treatment. Unfortunately, rifampin—present in nearly all regimens for both TB and NTM infections—complicates the concomitant use of antifibrotic therapy due to the high risk of acute liver failure.

Hepatotoxicity undoubtedly remains the primary reason why antifibrotic therapy should not be prescribed concurrently with antimycobacterial treatment. However, beyond this concern, both therapies are associated with significant side effects that may further challenge the continuous adherence to medication regimens. In Case #2, the patient developed antitubercular drug-induced anemia, requiring a red blood cell transfusion to manage the condition. Similarly, in Case #3, a suspected allergic reaction to pyrazinamide necessitated the discontinuation of this medication and its replacement with moxifloxacin.

A recent paper by Watanabe and colleagues reported a shorter survival time in subjects affected by *Mycobacterium avium complex* (MAC)-ILD compared to usual MAC cases. According to the authors, this finding possibly depends on the fact that treatment was not initiated in many patients due to the state of ILD, leading to a shortened survival time. Additionally, those affected by fibro-cavitary forms of MAC-ILD had a shorter survival time than the nodular-bronchiectatic patients [38].

Currently, there are no studies in the literature that directly evaluate the concomitant use of antifibrotic and antimycobacterial therapy. In cases of TB and cavitary or rapidly growing NTM infections, we recommend an approach that prioritizes antimycobacterial treatment based on our center’s experience. However, in slowly growing NTM infections, particularly in paucisymptomatic patients who are reluctant to undergo long-term (usually 12 months after culture negativization, typically a minimum of 14 months) antimycobacterial therapy due to concerns about the side effects of a three- or four-drug regimen, prioritizing antifibrotic treatment could be considered.

As previously reported by Shacor et al., it remains unclear whether the mycobacterium itself induces a pulmonary inflammatory state that subsequently leads to fibrosis and whether treating the mycobacterial infection alone would be sufficient to halt fibrosis progression, although its radiological presentation resembles a UIP pattern [31].

In conclusion, our case series highlights some novel findings regarding ILD patients with UIP pattern on chest HRCT and lung cavitary lesions. Firstly, the management of both diseases should be tailored case by case, as patients’ prognosis and treatment response can vary widely, as demonstrated by our cases. Additionally, the combined administration of both antifibrotic treatment and antimicrobial therapies for NTM-PD or TB presents a spectrum of tolerability that differs from patient to patient. Although antifibrotic and NTM/TB drugs have a narrow therapeutic index and are often associated with a high risk of significant side effects leading to treatment discontinuation, some patients can tolerate both treatments with manageable adverse effects. Another crucial point is the role of MDD to enhance the diagnostic accuracy; due to the wide number of differential diagnoses associated with the UIP pattern, as outlined in Table 1, and the variety of microorganisms responsible for cavitary lesions, MDD can play a crucial role in improving both diagnosis and treatment. This integrated approach can help guide the use of immunosuppressive/antifibrotic drugs alongside antimicrobial treatments, ultimately leading to more informed decision-making in managing these complex cases. Moreover, the well-known increased risk of ILD patients to develop cancer, as well as the ability of TB lesions to mimic tumors with necrosis areas, represent a further relevant diagnostic challenge. It also supports the role of MDD to identify the correct management of these patients according to the different treatments that tumors could require compared to ILDs with UIP and cavitary lesions of infectious origins. Overall, individuals affected by both ILDs with UIP pattern and cavitary lesions demand a personalized and multidisciplinary approach. The management of these cases should be evaluated on an individual basis, considering the potential administration of antifibrotic treatment and antimicrobial treatment for NTM/TB, with the goal of optimizing patient outcomes while minimizing drug-related side effects.

## Figures and Tables

**Table 2:** Demographic, clinical, microbiological, and radiological characteristics of the three patients.

	Sex/ Age *	Smoking Status	Timing of Diagnoses **	Clinical Presentation	ILD Pattern/ Diagnosis	NTM/TB Radiological Pattern	Biological Sample	Microbiology	ILD and NTM/TB Treatment	Patient Outcome
#1	M/76	Former	Three-year history of CTD-ILD before NTM infection	Massive hemoptysis, type 1 respiratory failure requiring hospital admission	Definite UIP CTD (RA)-ILD	Fibrocavitary	BAL	*M. kansasii*	Azathioprine rifampicin, azithromycin, and ethambutol	Alive
#2	F/82	Former	CTD-ILD prior to TB	Worsening of cough, dyspnea, and weight loss; type 1 respiratory failure requiring hospital admission	Definite UIP-CTD (RA)-ILD	Fibrocavitary	BAL	MDR Mtb	Amikacin, amoxicillin/clavulanate, meropenem, clofazimin, cicloserin, bedaquilin, and moxifloxacin	Died before completing therapy
#3	M/70	Former	concomitant diagnoses	productive cough and dyspnea on exertion	Definite UIP-IPF	Bronchiectatic	Induced sputum	Mtb	Nintedanib, rifampicin, isoniazid, ethambutol, pyrazinamide, (moxifloxacine)	Alive

* Age is considered at the time of the microbiological diagnosis; ** timing refers to the temporal relation between the microbiological and the ILD diagnosis; abbreviations: ILD: interstitial lung disease; UIP: usual interstitial pneumonia; CTD: connective tissue disease; RA: rheumatoid arthritis; MDR: multi-drug resistant; BAL: bronchoalveolar lavage; TB: tuberculosis; Mtb: *Mycobacterium tuberculosis*.

## Data Availability

All relevant data are within the manuscript. Raw data are accessible, if requested, from the National Institute for Infectious Diseases “L. Spallanzani” Library to the following e-mail address: biblioteca@inmi.it.
